# Assessing the use of unstructured electronic health record data to identify exposure to firearm violence

**DOI:** 10.1093/jamiaopen/ooae120

**Published:** 2024-11-04

**Authors:** Nicole Cook, Frances M Biel, Natalie Cartwright, Megan Hoopes, Ali Al Bataineh, Pedro Rivera

**Affiliations:** OCHIN Inc, Portland, OR 97228-5426, United States; OCHIN Inc, Portland, OR 97228-5426, United States; Department of Mathematics, Norwich University, Northfield, VT 05663, United States; OCHIN Inc, Portland, OR 97228-5426, United States; David Crawford School of Engineering, Norwich University, Northfield, VT 05663, United States; OCHIN Inc, Portland, OR 97228-5426, United States

**Keywords:** firearm violence, natural language processing, ambulatory care

## Abstract

**Objectives:**

Research on firearm violence is largely limited to people who experienced acute bodily trauma and death which is readily gathered from Inpatient and Emergency Department settings and mortality data. Exposures to firearm violence, such as witnessing firearm violence or losing a loved one to firearm violence, are not routinely collected in health care. As a result, the true public health burden of firearm violence is underestimated. Clinical notes from electronic health records (EHRs) are a promising source of data that may expand our understanding of the impact of firearm violence on health. Pilot work was conducted on a sample of clinical notes to assess how firearm terms present in unstructured clinical notes as part of a larger initiative to develop a natural language processing (NLP) model to identify firearm exposure and injury in ambulatory care data.

**Materials and Methods:**

We used EHR data from 2012 to 2022 from a large multistate network of primary care and behavioral health clinics. A text string search of broad, gun-only, and shooting terms was applied to 9,598 patients with either/both an ICD-10 or an OCHIN-developed structured data field indicating exposure to firearm violence. A sample of clinical notes from 90 patients was reviewed to ascertain the meaning of terms.

**Results:**

Among the 90 clinical patient notes, 13 (14%) notes reflect documentation of exposure to firearm violence or injury from firearms. Results from this study identified refinements that should be considered for NLP text classification.

**Conclusion:**

Unstructured clinical notes from primary and behavioral health clinics have potential to expand understanding of firearm violence.

## Introduction

Firearm violence in the United States is a major public health challenge with significant implications on health. The extent of the burden is markedly underestimated as International Classification of Disease (ICD) codes are largely limited to acute bodily trauma.^1–6^ The broader spectrum of firearm violence experiences that can lead to adverse physical and behavioral impacts, including witnessing or losing a loved one to firearm violence, is largely absent from firearm violence surveillance and research.^7^ The lack of data on firearm violence exposure hampers the ability of researchers and public health practitioners to fully frame and address the multifaceted health impacts of firearm violence.^8^

Electronic health records (EHR) are a promising potential source of data to enhance our understanding of firearm violence experiences and resultant injury.^9^ Clinical notes are stored as unstructured free text data within patient EHRs, and are a rich source of clinical context and patient and provider narrative. Natural Language Processing (NLP) may be used to build text classifiers to identify firearm exposure and injury, which can then be used to create a structured data element that identifies firearm exposure or injury (or neither). Methods to apply NLP to EHR data have generated interest across a broad range of health conditions, most notably chronic disease, social determinants of health, cancer, and mental health.^10–13^ To our knowledge, NLP has not been used to identify firearm injury in EHRs, though three studies on unrelated firearm topics provide guidance on key terms that are applicable to identify firearm violence experiences in clinical notes.^9^^,^^14^^,^^15^

To assess the feasibility of using NLP to identify exposure to firearm violence from clinical notes, our study searched for key firearm terms within a sample of EHR clinical notes from patients with exposure to firearm violence documented as an ICD code and/or in a customized data collection tool (SmartForm) implemented across hundreds of community-based health clinics which asked patients about exposure to firearm violence.^16^ The objectives of this study were (1) to identify if EHR clinical notes for patients with documentation of exposure firearm violence contain firearm keywords, and (2) to identify what firearm experiences, if any, are associated with these keywords. Both objectives are foundational activities in developing NLP models to identify patients with exposure to firearm violence from novel data sources.

## Methods

Our study used EHR data from the OCHIN (not an acronym) multistate network of community-based primary care and behavioral health ambulatory clinics. OCHIN offers a fully hosted, highly customized instance of Epic practice management and EHR solutions to more than 1300 care delivery sites across the United States.^17^ Most clinics in the OCHIN network provide care to medically underserved areas and populations.^18^

Investigators first identified all patients (n = 9,598) who had indication of exposure to firearm violence or injury in their EHR. Indication of firearm injury was determined by the presence of an ICD code in the patient’s EHR diagnosis or problem list from 01/01/2012—12/31/2022 (covering the timespan of available data). Indication of exposure to firearm violence was determined by a value of “recent” or “historical” in the SmartForm field “exposure to gun violence” any time from the implementation of the SmartForm on February 1, 2022, through the study end date of December 31, 2022. The SmartForm is a behavioral health trauma assessment tool that assesses exposures to multiple traumas. It was developed by an OCHIN behavioral health workgroup and integrated into the EHR in February 2022 to support trauma-informed care.^16^ The use of the SmartForm tool is available to mental and behavioral health providers and is used at their discretion.

There were 9,598 patients from 309 behavioral health clinics with known exposure to firearm violence. Of these, 9,350 had at least one clinical note; we did not limit the clinical notes by type of care (eg, primary care, behavioral health care, refill request). A text string search was performed among all clinical notes (n = 541,823) from these 9,350 patients for broad firearm violence terms (eg, firearm), gun-only terms (eg, gun), and shooting verbs (eg, shot) (see Supplementary Appendix) There were 5,489 distinct patients that had at least one clinical note that met the criteria of having at least one text string search term.

A random sample of 30 clinical notes from these 5,489 patients was chosen to represent each text string search category (broad, gun-only, and shooting), for a total of 90 sampled notes from 90 patients (patients and notes were distinct for each text string search category). These samples were reviewed by the primary author (N.C.) who extracted a short summary text with the relevant term(s) and created categories of the notes based on review. Two additional study team members (M.G. and F.B.) reviewed categorization of the summary text and confirmed anonymity of data.

Norwich University IRB deemed the study exempt.

## Results

The review of 90 random clinical notes included 61 (67.8%) patients with an ICD for firearm injury, 27 (30.0%) patients with a SmartForm field indicating exposure to firearm violence, and two (2.2%) patients with both an ICD and a SmartForm field indicating firearm injury and exposure to firearm violence. The distribution of firearm violence documented in the EHR record with either an ICD or a SmartForm field was similar to the total population, though there were slightly more patients with both an ICD and a SmartForm field in the random sample (2.2% vs .03%) (Table 1).

**Table 1.** Percent of patients (n = 5,489) and study sample (n = 90) with exposure to firearm violence documented with ICD code, SmartForm, or both

	ICD code	SmartForm^a^	ICD + SmartForm	Total
Total patients	66.4%	33.3%	0.3%	100.0%
Sample patients	67.8%	30.0%	2.2%	100.0%

a SmartForm = structured EHR data indicating exposure to firearm violence.

Among the 90 clinical patient notes reviewed, six clear categories emerged from the contextualization of the clinical notes (see Table 2). Our findings indicate that 13 clinical notes (14%) reflect exposure to firearm violence or injury from firearms; 36 clinical notes (40%) to firearm terms as part of patient assessment (eg, “do you have access to ‘guns’”); 20 notes (22%) refer to firearm terms in the context of anticipatory guidance and education (eg, “remove “guns” from the home”); and 16 notes (18%) include terms as part of treatment planning and care delivery (eg, immunization “shot,” “shooting” pain) (see Table 3).

**Table 2.** Category of firearms terms in clinical notes, with examples.

Category	Examples
Exposure to firearms	*She hears gunshots…* *Gunshot wound of the right lower leg.* *…suffered a GSW.* *Pt. was robbed at gunpoint.* *…he recalls his father’s friend sticking a gun in his mouth.* *history of gunshot.*
Assessment	*No access to firearms.* *No guns.* *Do you have any guns in the home? No* *Talks or writes about death, including writing suicide notes and talking about guns, knives or pills?* *Denies… no guns.* *Guns in the home. Locked up.*
Guidance and education	*Remove guns from the home.* *Make sure any guns in the home are locked up.* *Gun safety: education done.* *Keep opioids under lock and key. They are far more dangerous than blasting caps, and guns.*
Treatment planning and/or care delivery	*…shooting pain all the way down.* *Shot of Hepatitis A.* *Here for dmpa shot only.* *…cortisone shot in left knee*
Word “Shot” in a template phrase	*[Shot for Birth Control: Care Instructions.]*
“Gun” in a name	*Provider name includes gun (eg, Gunther)*
Other	*Shot a nailgun* *Snap shot includes current meds/allergies/problem list*

**Table 3.** Distribution of clinical notes (N = 90) by category of firearm terms^a^.

Category	%	N
Exposure to firearms	14	13
Assessment	40	36
Guidance and education	22	20
Treatment planning and/or care delivery	18	16
Word “Shot” in a template phrase	<5	< 10^b^
“Gun” in a name	<5	< 10^b^
Other	<5	< 10^b^
	100	90

a Categories are mutually exclusive.

b Counts <10 are suppressed for aggregate results.

## Conclusion

Exposure to firearm violence is common and associated with physical and behavioral health impacts.^19^^,^^20^ However, such data are not routinely collected as standardized data which limits their utility in surveillance and research. This small study found that EHR text strings within clinical notes contain contextual data on exposure to firearm violence. These novel data can leveraged for NLP development to support research on the prevalence of exposure to firearm violence and injury as well as the health sequelae following such exposure.^8^^,^^21^^,^^22^

Study findings provide important considerations for developing a labeled training set for NLP text classification. Specifically, proper names that include keywords (eg, “Gunther”), “shot” terms that are combined with immunization, birth control, or other injectable medications in the text string and “shooting” terms where “pain” is in the string without other reference to firearm terms will be removed. In addition, SmartPhrases and other template phrases pre-set in the EHR that include firearm terms as part of assessments or treatment planning that are not related to firearm injury or violence should be identified and removed. Finally, words, phrases and notes where the presence of a keyword does not represent firearm violence exposure or injury will be included in the labeled training set to improve performance.

Of note, the chart review conducted as part of this study was limited to patients with an ICD and/or patient assessment indicating exposure to firearm violence or firearm injury and represents a biased sample. Our study team’s next steps include (1) applying the above-mentioned refinements to keyword search terms, (2) analyzing refined keyword search findings in clinical notes where exposure to firearm violence is unknown, and (3) compiling a labeled training set for development of a machine learning NLP text classifier. Future work on larger samples should also examine differences in demographic, social, and clinical characteristics of patients identified with firearm exposure.

## Supplementary Material

ooae120_Supplementary_Data

## Data Availability

Aggregate data, qualitative codebooks, and statistical data analytic code (eg, R, SAS) may be shared upon request. OCHIN defines aggregate data as a dataset or data display that consolidates data from multiple individuals (eg, patients) and does not contain identifiers that can be used to identify individual patients. Patient level data will not be shared. The OCHIN Research Data Warehouse (RDW) includes patient-level data generated from multiple health systems across the OCHIN network; restrictions apply to the availability and re-release of patient-level data under organizational member agreements.
