# Supplementary material for: Assessing the use of unstructured electronic health record data to identify exposure to firearm violence
Source: JAMIA Open. 2024 Nov 4;7(4):ooae120. doi: 10.1093/jamiaopen/ooae120 (PMC11534176; doi:10.1093/jamiaopen/ooae120)
Supplement: ooae120_Supplementary_Data [file ooae120_supplementary_data.pdf]

Appendix 1. Search terms used to identify clinical notes likely to reference exposure to gun violence.

Broad gun violence terms:
'% GUN%' (with leading space)
'% FIREARM%'
'% PISTOL%'
'% HANDGUN%'
'% WEAPON%'

Gun-only terms:
'% GUN%' (with leading space)

Shooting verbs:
'% SHOT %' (leading and trailing spaces, excluding terms of "vaccine" and phrase "flu shot" in the text string);
'% GSW %'
'% GUNSHOT%'
'% SHOOTING %'.
